# Insights on the Effects of Heat Pretreatment, pH, and Calcium Salts on Isolation of Rare *Actinobacteria* from Karstic Caves

**DOI:** 10.3389/fmicb.2017.01535

**Published:** 2017-08-08

**Authors:** Bao-Zhu Fang, Nimaichand Salam, Ming-Xian Han, Jian-Yu Jiao, Juan Cheng, Da-Qiao Wei, Min Xiao, Wen-Jun Li

**Affiliations:** ^1^State Key Laboratory of Biocontrol and Guangdong Provincial Key Laboratory of Plant Resources, School of Life Sciences, Sun Yat-sen University Guangzhou, China; ^2^Medical Faculty of Kunming University of Science and Technology Kunming, China; ^3^Yunnan Institute of Microbiology, Yunnan University Kunming, China

**Keywords:** Sigangli Cave, rare *Actinobacteria*, heat pretreatment, pH, calcium salts

## Abstract

The phylum *Actinobacteria* is one of the most ubiquitously present bacterial lineages on Earth. In the present study, we try to explore the diversity of cultivable rare *Actinobacteria* in Sigangli Cave, Yunnan, China by utilizing a combination of different sample pretreatments and under different culture conditions. Pretreating the samples under different conditions of heat, setting the isolation condition at different pHs, and supplementation of media with different calcium salts were found to be effective for isolation of diverse rare *Actinobacteria*. During our study, a total of 204 isolates affiliated to 30 genera of phylum *Actinobacteria* were cultured. Besides the dominant *Streptomyces*, rare *Actinobacteria* of the genera *Actinocorallia*, *Actinomadura*, *Agromyces*, *Alloactinosynnema*, *Amycolatopsis*, *Beutenbergia*, *Cellulosimicrobium*, *Gordonia*, *Isoptericola*, *Jiangella*, *Knoellia*, *Kocuria*, *Krasilnikoviella*, *Kribbella*, *Microbacterium*, *Micromonospora*, *Mumia*, *Mycobacterium*, *Nocardia*, *Nocardioides*, *Nocardiopsis*, *Nonomuraea*, *Oerskovia*, *Pseudokineococcus*, *Pseudonocardia*, *Rhodococcus*, *Saccharothrix*, *Streptosporangium,* and *Tsukamurella* were isolated from these cave samples.

## Introduction

Caves provide a quasi-extreme environment for living organisms owing to relatively low organic nutrient input and lack of light (Pedersen, 2000). Some ‘sojourners’ such as crickets, spiders, olms and bats are, however, adapted to these unassumingly harsh environments through modifications in their body morphology and other physiological changes (Culver and Pipan, 2009). Unlike these organisms, the microscopic counterparts are abundantly present within the cave environment (Wu et al., 2015; Tomczyk-Żak and Zielenkiewicz, 2016), and they are involved in the dissolution and precipitation of karst minerals (Castanier et al., 2000). Despite many studies on the potential function and diversity of these microbes in the oligotrophic environments, our knowledge on cave microbial diversity and related bioactivities are still limited (Engel et al., 2004).

Earlier studies on caves indicated that bacteria and archaea constitute the majority of the microbial diversity (Barton and Jurado, 2007). Pyrosequencing analyses had determined phyla *Proteobacteria*, *Acidobacteria,* and *Actinobacteria* to be among the dominant taxa on cave environments (Pasić et al., 2010; Wu et al., 2015; Tomczyk-Żak and Zielenkiewicz, 2016). Among the major bacterial lineages, the phylum *Actinobacteria* are of special interest due to their versatile metabolic activities (Genilloud, 2014; Remenár et al., 2014). They are found ubiquitously in nature. Besides their role in biodegradation and production of ecologically important bioactive metabolites, they are also involved in biomineralization (Gillieson, 1996; Dhami et al., 2013). Based on the biotechnological significance of the phylum *Actinobacteria*, the basic aim of this work is to study the diversity of cultivable rare *Actinobacteria* in a karst cave located in Yunnan, China.

Though bacterial richness and diversity within specific environmental samples and their possible physiological role in nature can be established with NextGen sequencing techniques and other bioinformatics tools (Green and Keller, 2006), physiologies can only be verified with pure cultures (Leadbetter, 2003). It is, however, estimated that 99% of the existing microbes have not been cultivated yet (Whitman et al., 1998). It is therefore necessary to utilize various enrichment techniques or media to bring these uncultivated cells into cultures. Some of these techniques of culturing previously uncultivable soil bacteria have been reviewed by Pham and Kim (2012). In their review, major emphases are given upon the modification of growth conditions and use of new culturing methods. In the current, we try to explore the option of using a combination of enrichment techniques including heat-pretreatments of the samples, adjusting the isolation media into a pH gradient and supplementing the media with different calcium salts at different concentration. These techniques have already been established as an effective measure for isolation of diverse rare *Actinobacteria* (Alferova and Terekhova, 1988; Hayakawa, 2008; Lauber et al., 2009), but have not been exploited to determine the cultivable actinobacterial diversity in caves.

## Materials and Methods

### Site Description and Samples Collection

**FIGURE 1:**
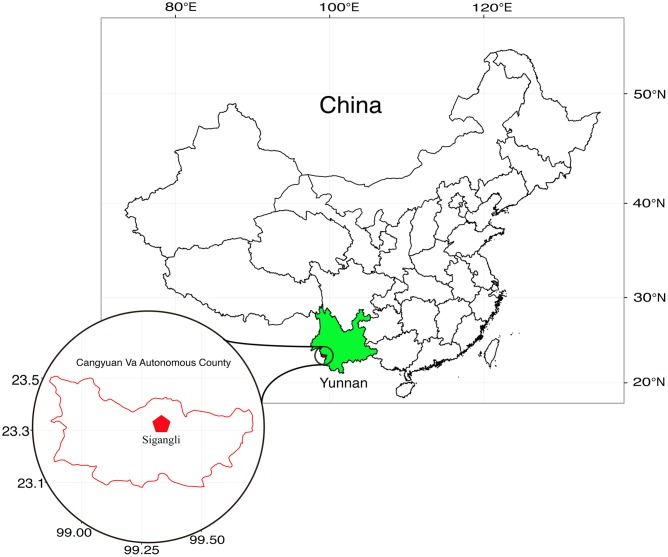
Geographical location of Sigangli Caves, Yunnan Province, China.

The Sigangli Caves, located in Cangyuan County, Yunnan Province, China, are part of a series of karst caves of the Yunnan–Guizhou Plateau formed from the dissolution of limestone and other calcareous rocks. The plateau, covering an area of over 1.3 × 10^5^ sq. km, was formed during tectonic shifts of Eurasian plate and is the center of karst area in East Asia (**Figure 1**).

Samples for isolation of *Actinobacteria* were collected from different part of the caves (23°32′ N, 99°33′ E). The samples (**Table 1**) include the hard rock forms (sedimentary rocks and cave coral, referred to here as Type 1 sample) and the weathered rock forms (saprolites, sand, debri and arene, referred to here as Type 2 sample). The samples were collected using sterile scalpels or spades and were transferred immediately in falcon tubes or Ziploc bags. These samples were then stored under a low temperature environment until processing for isolation.

**Table 1:** Description of the samples used for isolation.

Samples	Sample form	pH of samples	Sampling date	Sampling site	Coordinates
STRS01	Dark saprolite	7.8	30-03-2013	Sigangli	E 99.334′
SST6	Sedimentary rock	7.3			N 23.325′
SS16	Sandy soil	8.3			
SS19	Debris	8.0			
CS7	Arene	8.5	01-04-2013		
CST1	Cave coral	7.1			
YS5	Saprolite	7.5			
CS4	Stony and sandy soil	7.6	02-04-2013		
BS1	Dark saprolite	8.1	03-04-2013		

### Isolation and Preservation of *Actinobacteria*

A set of pretreatments and inoculation procedures adopted for the isolation of culturable *Actinobacteria* is listed in **Table 2**. Samples (2 g) were suspended in 20 ml sterile distilled water and kept in a rotary shaker (180 rpm, 28°C) for 1 h. The suspensions were serially diluted and aliquots of 100 μl of the diluted suspension was plated on freshly prepared agar media (in triplicates). The following isolation media were used: Humic acid-vitamin medium (HV) (Hayakawa and Nonomura, 1987); International *Streptomyces* Project (ISP) 5 medium (Shirling and Gottlieb, 1966); Cellulose-casamino acid (CC) medium (Yuan et al., 2017); Trehalose-proline agar (HP) (Li et al., 2014); Starch-Casien (SC) medium (Küster and Williams, 1964); B-4 medium (Boquet et al., 1973), and Water agar containing 11 g of agar per liter of water. Each of these media was supplemented with nystatin (50 mg L^-1^) and nalidixic acid (20 mg L^-1^) to prevent the growth of fungi and fastidious bacteria. Following incubation for 2 weeks at 28°C, all the colonies developed on the isolation media were counted. Depending on the isolation conditions and the media used, total colony forming units (CFUs) from each treatment were determined. Apart from the spore-forming strains, viabilility of the other vegetative cells were determined by subculturing on YIM 38 medium (Zhao et al., 2010). Heatmaps representing the distribution of these CFUs across the different physiological parameters were generated with R software (R Core Team, Vienna, Austria). The heatmaps are generated by applying ‘heatmap.2’ function of ‘gplots’ package. Further, morphologically distinct colonies were selected and purified on YIM 38 medium. The purified cultures were preserved as lyophilized cultures in skim milk and as glycerol suspensions (20%, v/v) at -80°C.

**Table 2:** Effects of physiological parameters on isolation of *Actinobacteria.*

S. no.	Physiological conditions for isolation	Pretreatment conditions	Samples for the study	Isolation media used
1	Sample pretreatment methods	(a) Fresh samples w/o pretreatment	SST6 (Type 1), SS19 (Type 2)	HV,
		(b) Air dried in room temperature for 2 weeks		ISP5,
		(c) Samples kept in oven at 40°C for 2 days		CC,
		(d) Samples kept in oven at 65°C for 4 h		HP,
		(e) Samples heated in oven at 110°C for 1 h		SC
		(f) Pretreatment e followed by c		
2	Effect of pH	Isolation media adjusted to pH 6, 7, 8, and 9	SS16 (pH 8.3),	CC,
			SS19 (pH 8.0),	HP
			YS5 (pH 7.5),	
			CS7 (pH 8.5),	
			CS4 (pH 7.6),	
			BS1 (pH 8.1)	
3	(a) Preference of calcium salts	(a) Isolation media supplemented with one of the three calcium salts CaCO_3_/CaCl_2_/(CH_3_COO)_2_Ca	CST1 (Type 1), STRS01 (Type 2)	B-4,
	(b) Concentration of the salts	(b) Concentration of each calcium salts adjusted to 0, 0.01, 0.1, and 1% (w/v)		HP,
				Water agar

### DNA Isolation, PCR Amplification, and Sequencing

Genomic DNA was extracted using TIANGEN^TM^ Genomic DNA purification kit according to manufacturer’s instructions. The DNA preparations were used as template for PCR amplification using the universal primers 27F: 5′-CAGAGTTTGATCCTGGCT-3′ and 1492F: 5′-AGGAGGTGATCCAGCCGCA-3′. PCR reactions were conducted using iCycler Thermal Cycler (Bio-Rad, USA Laboratories, Inc.) by applying the following conditions: initial denaturation at 94°C for 4 min; 30 cycles of denaturation at 94°C for 1 min, annealing at 55°C for 1 min, and extension at 72°C for 1 min, followed by a final extension at 72°C for 10 min. Amplified PCR products were verified on 0.8% agarose gel with 2 Kb DNA ladder (Fermentas) as a molecular size reference and sent for sequencing (Sangon Biotech, Shanghai, China).

16S rRNA gene sequences of the isolates were compared with the published 16S rRNA gene sequence database in EzBioCloud server (Yoon et al., 2017) on the basis of pairwise alignment. The strains were identified based on the sequence similarity to their closest homolog. Strains showing identity to bacterial phyla, other than *Actinobacteria*, were not reflected in the current study. Relative abundance of the *Actinobacteria* based on the number of strains from each genera were plotted into a scatter-plot using Microsoft Excel 2013.

### Nucleotide Accession Numbers

The partial 16S rRNA gene sequences of all the cultivated actinobacterial strains isolated during the study were deposited in GenBank with the following accession numbers: KX274728–KX274786, MF431270–MF431414 (Supplementary Table S1).

## Results

### Effects of Temperature, pH, and Calcium Salts on Isolation of *Actinobacteria*

**FIGURE 2:**
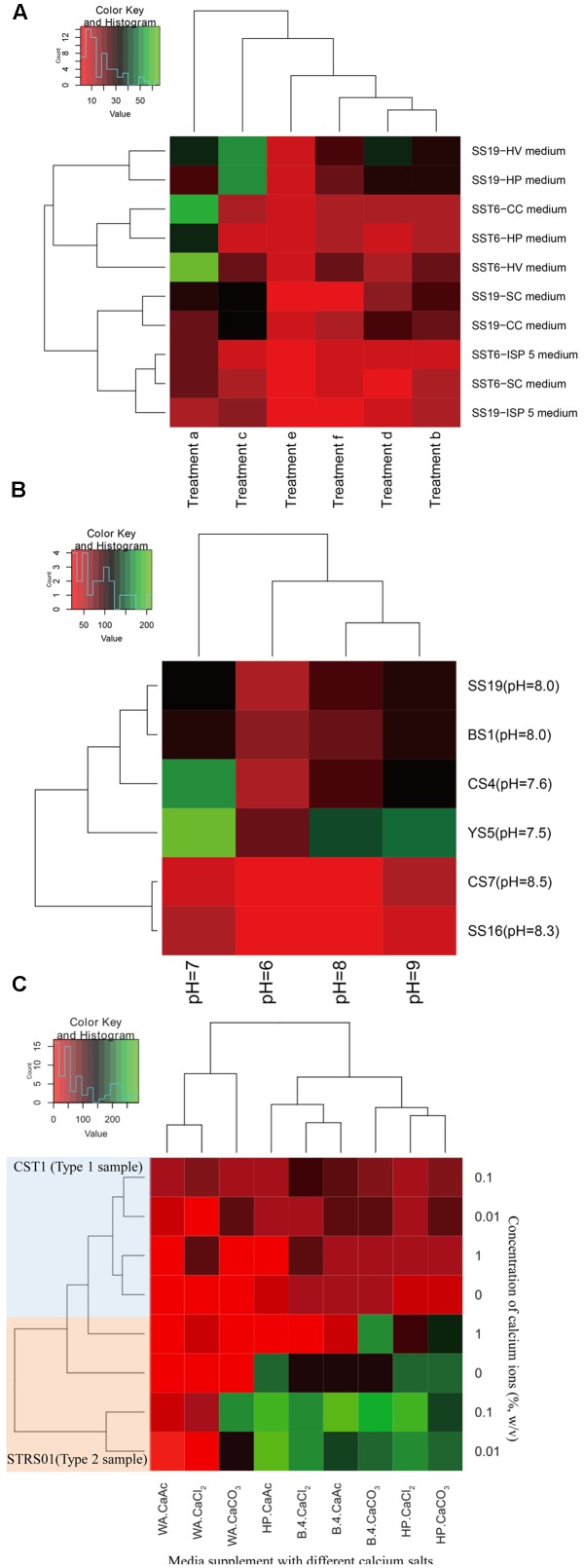
Heatmaps indicating the number of CFUs obtained after enrichments of samples collected from a karstic cave in Sigangli, China. The labels **(A–C)** represent the effects of temperature, pH, and calcium salts on the isolation of rare *Actinobacteria*, respectively. The heatmaps are generated in R software by applying ‘heatmap.2’ function in ‘gplots’ package.

**Figure 2** represents the effects of the different enrichment methods on the isolation of *Actinobacteria*. When heat pretreatments was used as the enrichment techniques (**Figure 2A**), more CFUs was determined in samples incubated at 40°C for 2 days prior to isolation (Treatment c) than in samples incubated at higher temperatures (Treatments d, e, and f). It is, however, interesting to note that samples kept at room temperature (Treatment b) yield lower CFUs than the one incubated at 40°C. Among the five media during this process, HV agar seems to be a better isolation medium (**Figure 2A**). When pH was used as the isolation criteria, it was determined that more CFUs were obtained in media adjusted to neutral pH, fewer in alkaline pH and least in circumneutral pH (**Figure 2B**). If the sample pH is taken into consideration, there is a gradual decrease in the number of CFUs with increase in sample pH, irrespective of the sample types. Lastly, considering the use of calcium salts in isolation, it was determined that CaCO_3_ yielded more *Actinobacteria* than (CH_3_COO)_2_Ca and CaCl_2_ (**Figure 2C**). However, it was not just the salt that is important, the concentration of each salt in the selection media also played an important role. In our case, it was determined that higher CFUs was determined when salt concentration was proportionately at lower concentration (i.e., 0.1 and 0.01%, w/v) than in its absence or at high concentration.

### Relative Abundance of Rare *Actinobacteria*

**FIGURE 3:**
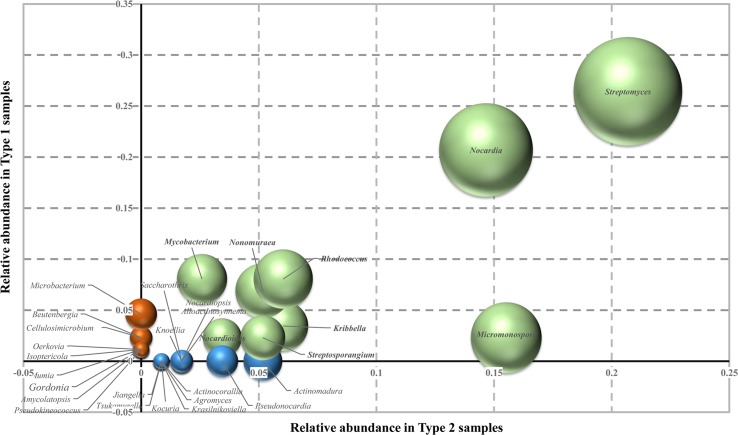
Relative abundance of rare *Actinobacteria* on the two types of cave samples used on this study. Values on the axes represented the relative abundance of each genus on the different samples. Green spheres indicate actinobacterial genera present in both the sample types; red, genera isolated from Type 1 samples only; and blue, genera found only in Type 2 samples. Sizes of the sphere quantify the relative number of strains of each genera. The scatter-plot is generated using Microsoft Excel 2013.

Of these total colonies observed, morphologically distinct colonies were further selected, subcultured, and preserved. These include 87 isolates from Type 1 samples and 117 from the Type 2 samples. Sequence analysis of 16S rRNA gene indicated that the strains from the Type 1 samples were distributed to 20 genera in 14 families of the phylum *Actinobacteria*, while the strains isolated from Type 2 samples were distributed to 21 genera and 16 families. The relative abundance of the strains is represented in **Figure 3**, and the 16S rRNA gene sequence profile listed in Supplementary Table S1. Besides the most abundant genus *Streptomyces*, the rare actinobacterial genera *Nocardia* and *Rhodococcus* were relatively abundant in both the sample types (18 and 7 strains respectively in Type 1 samples, and 17 and 7 strains in Type 2 samples). While the genus *Micromonospora* was relatively more abundant in Type 2 samples (19 strains, as compared to 2 in Type 1 samples), the genus *Mycobacterium* was more in hard rock (7) than in the weathered rock samples (3). Other rare genera that were common to both the sample types include *Jiangella*, *Kribbella*, *Nocardioides*, *Nocardiopsis*, *Nonomuraea,* and *Streptosporangium*. Apart from these common genera, few rare actinobacterial genera were restricted to only one particular sample type. The genera *Amycolatopsis*, *Beutenbergia*, *Cellulosimicrobium*, *Gordonia*, *Isoptericola*, *Microbacterium*, *Mumia*, *Oerskovia,* and *Pseudokineococcus* were isolated only from Type 1 samples while the rare genera *Actinocorallia*, *Actinomadura*, *Agromyces, Alloactinosynnema, Knoellia,* *Kocuria, Krasilnikoviella*, *Pseudonocardia*, *Saccharothrix,* and *Tsukamurella* were isolated from Type 2 samples.

## Discussion

Karstic caves are characterized by low stable temperature (10–15°C), relatively high humidity (90–100%) and total darkness or low level of light, and are often mystical and inaccessible for study. Above that, caves usually constitute a oligotrophic ecosystem with total organic carbon of less than 2 mg/L (Tomczyk-Żak and Zielenkiewicz, 2016). Despite the oligotrophic condition, the average number of microorganisms in this ecosystem have been estimated to be in the range of 10^6^ cells/g of rock (Barton and Jurado, 2007). Despite the estimated large number of bacterial cells in cave rocks, they could isolate around 400 bacteria belonging to phyla *Proteobacteria*, *Firmicutes,* and *Actinobacteria* from a sample of Lechuguilla Cave, New Mexico. Of these strains, nearly 40 strains are assumed to be previously uncultivated species indicating that the diversity of microbes within the caves is impressive (Barton and Jurado, 2007). In another study in Kartchner Caverns, Arizona, 90 unique isolates belonging to *Proteobacteria*, *Firmicutes,* and *Actinobacteria* were isolated, but these bacteria have 16S rRNA gene sequence similarity profiles to known bacteria (Ikner et al., 2007).

Among these group of bacteria, class *Actinobacteria*, because of its different versatile morphology, were detected and isolated from even the most extreme of environments (Mohammadipanah and Wink, 2016). The main mechanism for the survival of these actinobacteria in these environments is through the formation of different types of spores. Most actinobacterial spores are developed either endogenously (e.g., *Dactylosporangium*, *Thermoactinomyces*) or exogenously (e.g., *Streptomyces*) in response to environmental stress (Kalakoutskii and Agre, 1976). While in karst environments, the presence of minerals, particularly different forms of calcium rocks, trigger sporulation in many *Actinobacteria* (Kalakoutskii and Agre, 1976). These spores usually remain in dormant state with minimum respiration, but could be made to germinate in defined media by providing an energy source (Salas et al., 1983). In most case of germination, a mild stimuli is required either in the form of heating or supplying germinants that stimulate the disruption of spore cortex (Warth and Strominger, 1972; Ensign, 1978). In our effort to select and isolate diverse *Actinobacteria*, we considered three sample enrichments methods involving chemical and physical treatments as discussed below.

Among the physical enrichments method for actinobacterial isolation, air drying, dry heating, moist incubation, and desiccation have been found to be an effective for selection of spore-forming rare *Actinobacteria* (Hayakawa, 2008). Air-drying of soil at 120°C for an hour is usually preferred for isolation of genera *Dactylosporangium*, *Microbispora* and *Streptosporangium*, while limiting the growth of streptomycetes (Jiang et al., 2016). Similarly, air-drying at 100°C for 15 min have been used effectively for isolation of *Actinomadura* (Jiang et al., 2016). Air-drying the sample at an ambient temperature for a week preferentially select *Herbidospora* among other bacteria (Jiang et al., 2016). Genus *Micromonospora* were selectively isolated by pretreatment of samples at 55–65°C for 30 min (Jiang et al., 2016). An effective method proposed for isolation of rare *Actinobacteria* involved air drying at 80°C for 2 h (Goodfellow, 2010). Preferential selection of several rare *Actinobacteria* on heat treatments might be related to the spore-forming capability of several groups of *Actinobacteria* (Jiang et al., 2016). In the current study, we considered to pretreat our samples by air-drying at room temperature, 40, 65, and 110°C for different time intervals for effective isolation of diverse rare actinobacteria (**Table 2**). An advantages of air-drying is that many *Actinobacteria* produces spores, and that dry spores have low respiration rate and can survive for longer period of time. During this period of low level of endogenous respiration, the spores did not germinate, but can be germinated readily when a defined medium with organic energy source is provided (Ensign, 1978). During our study, four cells in the heatmap representation indicated CFUs’ count in the range of 50 or above (**Figure 2A**). These highest OTUs were represented in samples treated at low ambient temperatures. The levels of CFUs was found to decrease with increased pretreatment’s conditions. Least CFUs were obtained in the samples pretreated at 110°C for 1 h. However, if the pretreatment at 110°C for 1 h is accompanied by incubation at 40°C for 2 days, the numbers of sporulating cells that survived the heat treatment increased as indicated in the **Figure 2A**. This finding could be related to the study of Lapteva et al. (1976) whereby the authors suggested that the germination inhibitors produced in the spores due to heating were neutralized during subsequent incubation at lower temperature. If a comparative analyses were made between the number of CFUs obtained and the strains cultured during our study, positive correlation could be established between the number of spore-forming *Actinobacteria* obtained and the temperature used for pretreatment. While no strict thermophilic *Actinobacteria* such as members of the genera *Thermomonospora*, *Dactylosporangium,* etc. were isolated during our study, a fair number of rare *Actinobacteria* with aleurispores (*Micromonospora*), arthrospores (*Actinomadura*), sporangiospores (*Streptosporangium*), or other spore-bearing structures (*Amycolatopsis* etc.) were obtained. However, in addition to these *Actinobacteria*, endospore-forming non-*Actinobacteria* such as *Bacillus* strains were also obtained during the isolation process. The co-occurrence of these bacteria are, however, found to be very less as confirmed by our preliminary sequencing analysis of the 16S rRNA gene (data not shown).

*Actinobacteria* are capable of growing under selective conditions of pH or salinity (Mohammadipanah and Wink, 2016). It is because pH of soil strongly influence the biomass, activity and composition of the microbial community, and therefore pH in the isolation media provide a selective pressure for the growth of bacteria (Bååth, 1996; Matthies et al., 1997; Rousk et al., 2009). Unlike fungi which grow preferentially in acidic and moist condition, most *Actinobacteria* showed optimum growth on slightly alkaline condition (Kontro et al., 2005; Lewin et al., 2016). The isolation of strictly acidophilic *Actinobacteria* like the genus *Streptacidiphilus* (Kim et al., 2003; Cho et al., 2008; Golinska et al., 2016) from diverse ecosystems have provided a platform for isolation of *Actinobacteria* under acidic environments, in addition to the normally preferred slightly alkaline condition. Considering the wide range of pH on which *Actinobacteria* can dwell with, we considered to compare the CFU’s count under a gradient of pH range of isolation, despite all the sample pHs being in the range of 7.5 to 8.5 (**Table 2**). During the current study, more actinobacterial CFUs were detected in neutral pH (**Figure 2B**). This may be because of the easy maintanence of cell’s cytoplasmic pH at close to neutrality (Kontro et al., 2005). While the actinobacterial CFUs did not dwindle much in alkaline pH, it had the least count in acidic isolation media. This finding could also be related with the findings of Rousk et al. (2010) whereby the relative abundance of *Actinobacteria* was not affected by soil pH, but rather depended on the isolation condition (Lauber et al., 2009).

*Actinobacteria* often colonize the rock walls of caves. In a study on biogeochemical role of *Actinobacteria* in Altamira Cave (Spain), *Actinobacteria*-coated spots on the cave walls was found to uptake carbon dioxide gas which is available in abundance in cave (Cuezva et al., 2012). This uptake gas is used by the bacteria to dissolved rock and subsequently generate crystals of calcium carbonate (Cañaveras et al., 2001). While its role in biomineralization is plausible, calcium ions do play specific role in various spore-forming microorganism as well. While measuring the metal ion content in five *Streptomyces* strains (Salas et al., 1983), the level of calcium was found to be higher in dormant spore than in the vegetative cells. Calcium is mostly found as a complex with dipicolinate and this complex could be acting as secondary stabilizing agent for the spore against environmental stresses (Moir and Smith, 1990). However in the presence of suitable germinants, the spore release the calcium-dipicolinate complex from the core to initiate the process of spore germination (Moir, 2003). One process through which the complex acts is by initiating cortex degradation through structural modification of the peptidoglycan (de Vries, 2004). The use of calcium carbonate in pretreatment for selective isolation of *Actinobacteria* (El-Nakeeb and Lechevalier, 1963; Alferova and Terekhova, 1988) may be related with the spore formation in *Actinobacteria*. On the other hand, calcium chloride, when added to isolation media, was found to stimulate the growth of a rare heterotrophic *Actinobacteria*, *Sporichthya* (Suzuki et al., 1999). We, therefore, considered to compare the effect of supplementation of three calcium salts in the isolation media including CaCO_3_ and CaCl_2_. During our study, all three calcium salts facilitated the isolation of *Actinobacteria* (**Figure 2C**). This finding is also in congruence with the finding of Chen et al. (2016) whereby the actinobacterial community structures showed significant correlations with calcium. Warth and Strominger (1972) have determined that germination of bacterial spore required a optimum concentration of approximately 10 mM calcium ions. This observation is similar with our findings where a lower concentration of calcium ions (0.1% or ∼10 mM) provide more CFUs than higher (1%, w/v or ∼0.1 M) or in the absence of calcium salts (**Figure 2C**). Among the two types of samples, more CFUs were observed in Type 2 than in Type 1 samples. The reason could be implicated on the lower cell concentration on the surface of hard rock (Barton and Jurado, 2007).

In all the above cases, isolation media play the key role for providing the favorable condition for isolation and growth of rare *Actinobacteria*. It is therefore important to use isolation media that preferentially isolate different group of rare *Actinobacteria* and select/design set of media with different components to maximize our chance for isolation of unique and other rare *Actinobacteria* (Tiwari and Gupta, 2013), lest *Actinobacteria* will be at competitive disadvantages on the solid media against the fastidious bacteria and fungi that usually occupy a larger living space. In the current study, we had selected seven isolation media that have been found effective in isolation of *Actinobacteria*. Among them, HV agar with/without chemical supplements had been used efficiently by Hayakawa’s group for isolation of many rare *Actinobacteria* including strains of genera *Actinokineospora*, *Actinomadura*, *Actinoplanes*, *Actinosynnema*, *Catenuloplanes*, *Cryptosporidium*, *Dactylosporangium*, *Geodermatophilus*, *Herbidospora*, *Kineosporia*, *Microbispora*, *Micromonospora*, *Microtetraspora*, *Nonomuraea*, *Spirilliplanes*, *Sporichthya*, *Streptosporangium,* and *Virgosporangium* (Hayakawa, 2008). It may be the wide applicability of this media in isolation of different group of rare *Actinobacteria*, that we are able to found more CFUs in this medium (**Figure 2A**). While SC and ISP media were introduced for the isolation of mycelial-producing *Actinobacteria* particularly genus *Streptomyces* (Küster and Williams, 1964; Shirling and Gottlieb, 1966), B-4 media was established to be good for isolation of *Actinobacteria* precipitating calcium carbonate crystals (Boquet et al., 1973). The large amount of *Steptomyces* among our isolates could be correlated with the use of ISP and SC during our isolation (Supplementary Table S1). The media CC and HP were especially designed in our laboratory to isolate rare *Actinobacteria* that could utilize complex energy sources (Li et al., 2014; Yuan et al., 2017). Among these two media, HP was more efficient than CC in giving larger CFUs count (**Figures 2A,C**). The reason behind the larger CFUs in HP could not be ascertained from the current study, however, it is possible that degradation of cellulose required complex enzyme-system and that many *Actinobacteria* were not able to use cellulose as their energy sources. On the other hand, water agar, which is found to stimulate growth of spore-forming microorganisms, was not effective during our study for the growth of rare *Actinobacteria* (**Figure 2C**). Inhibition of certain rare actinobacterial strains by the preferential treatments, however, cannot be completely ruled out.

In the karstic caves, the primary production usually depends on chemoautotrophic bacteria (Sarbu et al., 1996). Recent studies have, however, revealed that considerable input of organic matter could support the growth of heterotrophic bacteria including *Actinobacteria* (Arroyo et al., 1997; Groth and Saiz-Jimenez, 1999). These findings instigated the study on diversity of *Actinobacteria* in several caves located around the world such as Niu Cave (Zhou et al., 2007), Pajsarjeva jama (Pasić et al., 2010), Wind Cave (Chelius and Moore, 2004), Kartchner Caverns (Ikner et al., 2007), Altamira Cave (Cuezva et al., 2009), and Altamira and Tito Bustillo Caves (Schabereiter-Gurtner et al., 2002). Studies of Shabarova and Pernthaler (2010) have resulted in the isolation of *Actinobacteria* belonging to the genera *Arthrobacter*, *Blastococcus*, *Curtobacterium*, *Kribella*, *Micrococcus*, *Nocardia*, *Promicromonspora*, *Pseudonocardia*, *Rhodococcus,* and *Streptomyces*. Unlike the above study, significant diversity of rare *Actinobacteria* were observed in the present study. These *Actinobacteria* were affiliated to genera *Actinocorallia*, *Actinomadura*, *Agromyces*, *Alloactinosynnema*, *Amycolatopsis*, *Beutenbergia*, *Cellulosimicrobium*, *Gordonia*, *Isoptericola*, *Jiangella*, *Knoellia*, *Kocuria*, *Krasilnikoviella*, *Kribbella*, *Microbacterium*, *Micromonospora*, *Mumia, Mycobacterium, Nocardia*, *Nocardioides*, *Nocardiopsis*, *Nonomuraea*, *Oerskovia*, *Pseudokineococcus*, *Pseudonocardia*, *Rhodococcus*, *Saccharothrix*, *Streptosporangium,* and *Tsukamurella*. The presence of genera *Micromonospora*, *Nocardia,* and *Rhodococcus* as the dominant rare *Actinobacteria* in our study was consistent with other related studies (Arroyo et al., 1997; Zhou et al., 2007; Valme et al., 2010).

Despite the isolation of varied actinobacterial groups after applying a set of pretreatments and modification of isolation media, our study suffers from few limitations. Firstly, the physicochemical parameters of the sampling site and the co-existence of different minerals/metals were not measured during the study. Lack of these data prevent us from indirect establishment of the interrelationship between the occurrence of different actinobacterial groups and their physiological roles in cave. Secondly, the study was limited to isolation of culturable *Actinobacteria*. As such, we could not equally verify if the applied methods were effective to deselect non-*Actinobacteria*. It is also equally possible that certain culturable rare *Actinobacteria* particularly non-spore formers were deselected due to the stressors provided in our pretreatments. Lastly, the isolation methods have not been replicated in other karst environments or any other habitats. A study on the total microbial composition using NextGen sequencing could provide an idea of the effectiveness of the isolation method. However, it can certainly be stated that the methods provided above proved effective for the isolation of many rare *Actinobacteria*, comprising of both spore-formers (e.g., *Actinocorallia*, *Alloactinosynnema*, *Jiangella*, *Oerskovia* etc.) and non-spore formers (such as *Agromyces*, *Beutenbergia*, *Cellulosimicrobium*, *Gordonia*, *Isoptericola*, *Kocuria*, *Tsukamurella*).

## Author Contributions

W-JL, B-ZF, and JC designed research and project outline. JC, M-XH, MX, and NS performed isolation, deposition, and identification. B-ZF, J-YJ, and D-QW contructed the heatmap and other related bioinformatic plots. B-ZF, NS, MX, and W-JL drafted the manuscript. All authors read and approved the final manuscript.

## Conflict of Interest Statement

The authors declare that the research was conducted in the absence of any commercial or financial relationships that could be construed as a potential conflict of interest.
